# Vexations

**Published:** 1846-12

**Authors:** 


*Vexations.*—It is a trite saying, that every pursuit in life has its peculiar sources of annoyance. Editors have an advantage over others, in the facilities of giving publicity to their complaints. Hence perhaps is it, that the vexations of editorship have become a proverb. We have occupied the editorial chair sufficiently long to have realized that he would commit a capital *faux pas* who should select it as a place for indolent repose, or for the acquisition of philosophical equanimity. We have a short catalogue of little 'vexations' occasioned mostly by the thoughtlessness of the patrons of periodical literature, which we will not publish, lest some of our friends may think we intend to be personal; but we will confine our remarks to one only.

Respected Reader and Subscriber! do your own thoughts at once suggest the topic to which we allude? Reflect for an instant: have you remitted according to "terms," and our gentle hints, the amount due for your subscription? If so, we congratulate you upon the smiles of an approving conscience as you peruse this article. But, alas! we have before us demonstrative evidence that *all* our readers cannot enjoy the satisfaction of feeling that our remarks have no personal application to themselves. Our subscription list discloses the names of not a few delinquents. Gentlemen! indulge us with a few words of expostulation. We have pledged ourselves under any circumstances to labor gratuitously for your advantage. Is it reasonable, on your part, to insist that we shall ourselves pay the little tax which the journal costs you? This we must do if you persist in your neglect, for the work is published under a guaranty from ourselves, and associates, that every subscription shall be paid before the expiration of the year. We can readily understand your reflections: you say to yourselves, in excuse for your reluctance to quit possession of the attractive bank note, 'other subscribers will probably pay, and my subscription among some hundreds, will not be worth thinking of.' Other claims too, are perhaps more urgent because nearer home, and we cannot dun *viva voce.* But consider that you are only one, of many, who omit paying for the same reasons, and while it is a small matter individually to you, it is, in the aggregate, a serious *vexation* to us. While we cheerfully give you the benefit of our labor without asking reward therefor, and are willing to make pecuniary sacrifices, should they be necessary to sustain the journal—as sustained it will be, life and health permitting—we feel that is unjust, and we will add unkind to us, on the part of subscribers, not to contribute with alacrity the trifle which we are entitled to claim from them for the benefit of those who publish the work. But we have said enough—the particular readers to whom these remarks will come home, by this time are doubtless filled with remorse at the consciousness of their grievous sin of omission. Do not, we pray you, feel too badly. It is not too late too redeem your errors; and we promise you full absolution for this past offence, provided you include with your subscription, *remitted without delay*, that of a new patron procured through your agency.

Our readers will perceive that this article is ostensibly a homily upon the vexations of editorship. It would perhaps be more dignified to keep oneself independent of all the pecuniary concerns of journalizing. We notice that some of our contemporaries are particular to advertise that all communications on this subject must be addressed exclusively to the publisher. Our enterprise, however, is purely a professional one, and for the sake of its objects the responsibility of it rests with those of the profession who have originated it. We cannot, therefore, in justice to ourselves, and others, be too fastidious to perform the disagreeable duty of investigating, from time to time, our subscription list. But we will not end with complaining. In truth there is much connected with our editorial undertaking to gratify and encourage. Our patrons increase, and everything promises all the success that we have a right to claim. We do not continue our labors with a complaining spirit, but with increased interest and satisfaction.—But enough of talking of ourselves. We promise, indulgent readers, not to introduce the subject again for a long while,—*provided our delinquent subscribers pay their dues.*

